# A multistage approach to blood conservation in a Jehovah's Witness patient undergoing redo aortic arch surgery

**DOI:** 10.1186/1749-8090-10-S1-A99

**Published:** 2015-12-16

**Authors:** Rahul Basu, Surendra Naik, Mohammad Shafaat, John Lu

**Affiliations:** 1 Trent Cardiac Centre, City campus, Nottingham University Hospitals NHS Trust, Hucknall Road, Nottingham NG5 1PB, UK

## Background/Introduction

49 year old woman with Marfan's syndrome previously treated with aortic root replacement and resuspension of aortic valve for type A dissection. Now presented with progressive dilating 6.3 cm arch aneurysm requiring redo surgery.

## Aims/Objectives

To describe a multi-stage approach of patient blood management in a Jehovah's Witness undergoing redo aortic arch surgery.

## Method

Preoperative phase

Baseline ferritin, transferrin, B12, folate and TFT levels.

Intravenous iron 1000 mg as single dose 3-5 weeks before planned surgery.

Epoetin alfa (600 units per kg) at weekly intervals starting 3 weeks before surgery and the 4th dose given day before surgery.

Hb improved from 119 to 152g/l.

Perioperative phase

Predonation of 2 units blood at induction.

Usage of aprotinin.

Near normothermic CPB at 30°C.

Usage of Hemosep^® ^device to recover blood spilled during surgery. This preserved platelets, white and red cells for subsequent transfusion.

Postoperative phase

Hb 103g/l immediately post-op but fell to 64g/l over 24h. Therefore ventilated electively for 2 days giving IV iron and erthropoietin at day 1 and 4 to enhance erythropoiesis.

Patient extubated when Hb 79 g/l.

## Results

Patient had no re-exploration for bleeding or tamponade. She had a period of CVVH for renal failure and IV antibiotic for chest infection.

She was transferred to the ward on day 9 and discharged home on day 13. Subsequent follow up at week 1 and 6 post discharge showed the patient doing well.

## Discussion/Conclusion

Meticulous multidisciplinary individualised patient blood management can result in safe and excellent outcome in a Jehovah's Witness patient undergoing major redo aortic surgery.

## Consent

Written informed consent was obtained from the patient for publication of this abstract and any accompanying images. A copy of the written consent is available for review by the Editor of this journal.

